# Iron Loss and Temperature Rise Analysis of a Transformer Core Considering Vector Magnetic Hysteresis Characteristics under Direct Current Bias

**DOI:** 10.3390/ma17153767

**Published:** 2024-07-31

**Authors:** Minxia Shi, Teng Li, Shuai Yuan, Leran Zhang, Yuzheng Ma, Yi Gao

**Affiliations:** 1 Key Laboratory of Ultra-Weak Magnetic Field Measurement Technology, Ministry of Education, School of Instrumentation Science and Optoelectronics Engineering, Beihang University, Beijing 100191, China; shiminxia@buaa.edu.cn (M.S.); 2423660154@buaa.edu.cn (T.L.); 21myz@buaa.edu.cn (Y.M.); gaoyi0509@163.com (Y.G.); 2 Zhejiang Provincial Key Laboratory of Ultra-Weak Magnetic-Field Space and Applied Technology, Hangzhou Innovation Institute, Beihang University, Hangzhou 310051, China

**Keywords:** vector magnetic properties, DC-biased field, magnetic characteristics measurement, FEM analysis, temperature rise

## Abstract

Direct current (DC) bias induced by the DC transmission and geomagnetically induced current is a critical factor in the abnormal operation of electrical equipment and is widely used in the field of power transmission and distribution system state evaluation. As the main affected component, the vector magnetization state of a transformer core under DC bias has rarely been studied, resulting in inaccurate transformer operation state estimations. In this paper, a dynamic vector hysteresis model that considers the impact of rotating and DC-biased fields is introduced into the numerical analysis to simulate the distribution of magnetic properties, iron loss and temperature of the transformer core model and a physical 110 kV single-phase autotransformer core. The maximum values of ***B***, ***H*** and iron loss exist at the corners and T-joint of the core under rotating and DC-biased fields. The corresponding maximum value of the temperature increase is found in the main core limb area. The temperature rise of the 110 kV transformer core under various DC-biased conditions is measured and compared with the FEM (Finite Element Method) results of the proposed model and the model solely based on the magnetization curve ***B***||***H***. The calculation error of the temperature rise obtained by the improved model is approximately 3.76–15.73% and is much less than the model solely based on magnetization curve ***B***||***H*** (approximately 50.71–66.92%).

## 1. Introduction

Transformers are essential in power transmission and distribution systems because their performance directly affects the security and stability of power grids. As long as a DC current appears in the transformer winding, the DC bias leads to a shift and an oversaturated magnetization state of the transformer core. The primary causes of DC bias in transformer windings are ground currents generated by DC transmission and geomagnetically induced currents (GICs) caused by magnetic storms [1,2,3]. Considering the extensive scale and long distances of high-voltage transmission lines, the surface potential induced by magnetic storms could result in a significant GIC amplitude and a wide-ranging influence on the power grid [4,5]. Additionally, large power transformers, especially those with single-phase ultrahigh voltage (UHV), are highly sensitive to DC magnetic flux, and the excess temperature and vibration rise of the iron core caused by the DC bias may lead to irreparable damage to the transformer [6,7].

In the study of DC magnetic bias, finite element analysis (FEM) is widely used in the simulation of magnetic parameters, loss and the temperature distribution in the transformer core [8,9]. The scalar magnetic properties of the AC (Alternating Current) magnetic field are commonly considered in FEM calculations of the transformer core performance under DC-biased conditions [10]. However, the rotating magnetic field and the related vector magnetic properties, which have a significant impact on the T-shaped interfaces and corners of the core, cause a non-negligible deviation of the loss and temperature distribution simulation [11,12]. The normal rotating magnetic field was studied under AC excitation, and vector magnetic characteristics were fitted by the E&S (Enokizono & Soda) model [13], vector Jiles–Atherton (J–A) model [14,15], vector Preisach model [16,17], etc. To describe the impact of the frequency and magnetization history on the vector magnetic properties, M. Enokizono and N. Soda proposed a differential E&S vector hysteresis model by combining the Chua-type model with the reluctance tensor model [18]. H. Shimoji proposed an integral E&S vector hysteresis model to achieve a better convergence [19,20]. Taking the eddy current effect into consideration, N. Kunihiro proposed an E&S dynamic vector hysteresis model and provided a description of the distortion phenomenon of the ***B*** locus [21,22]. The situation of a DC bias combined with a rotating magnetic field has been preliminarily studied [9,23]. However, the conventional vector magnetic model is rarely applied to a physical transformer core in DC-biased situations, and the corresponding additional local core losses and overheating effects, considering the hysteresis and eddy current effects caused by DC-biased and rotating fields, are rarely discussed.

In this study, an enhanced vector magnetic model considering the hysteresis and eddy current effects under a DC-biased situation was established and introduced into a loss and temperature distribution study of the physical transformer core. First, an experimental setup was established, and the corresponding magnetic properties under rotating and DC-biased fields were measured. Subsequently, the iron losses under various rotating and DC-biased fields were analyzed by considering the magnetic domain movement. Furthermore, an improved magnetic model that considers the impact of rotating and DC-biased fields was introduced into the simulation of the distribution of magnetic field, loss and temperature of the transformer core. Finally, the simulated results calculated using the proposed model and the magnetization curve were compared with the measured results of the temperature rise of a physical 110 kV single-phase autotransformer.

## 2. The Measurement of Rotating Magnetic Properties

### 2.1. The Measurement Setup

**Figure 1:**
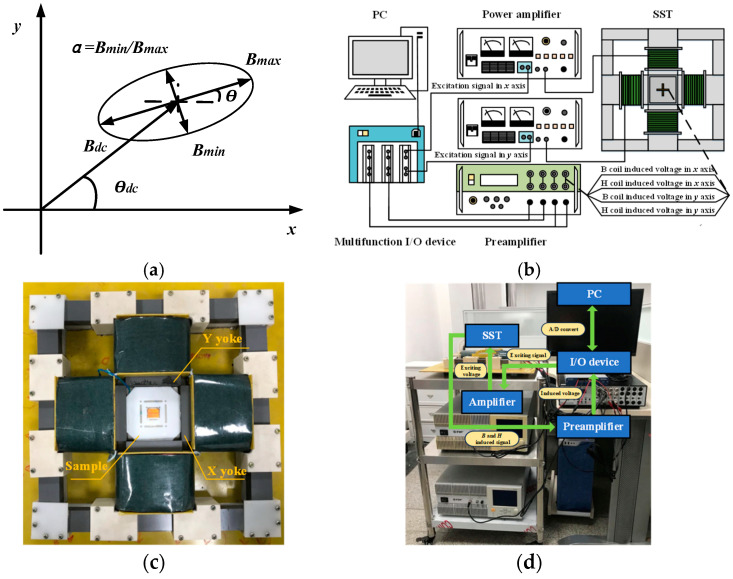
The desired field and the feedback measurement system: (**a**) Desired field. (**b**) Schematic of the experimental measurement system. (**c**) The physical single sheet tester (SST). (**d**) The physical measurement system.

The desired rotating magnetic field and the DC-biased field to be measured are shown in Figure 1a. The rotating magnetic field is defined by three parameters: the maximum value of the rotating magnetic field *B_max_*, the incline direction angle *θ*, and the ratio *α*, which is the ratio of the minimum value *B_min_* to the maximum value *B_max_*, as follows: *α* = *B_min_*/*B_max_*. *B_min_* is the minimum value at the ***B*** locus. The DC-biased field is characterized by two parameters: the amplitude *B_dc_* and the direction *θ_dc_*. The directions of the rotating magnetic field and DC-biased field are counted from the *x*-axis when *θ* and *θ_dc_* are set as zero. The *x*-axis means the rolling direction and the *y*-axis means the traverse direction. In this study, a feedback measurement system for the rotating and DC-biased fields’ excitations and tests was established, as shown in Figure 1b. The system is composed of the single sheet tester (SST), the preamplifier (Krohn-Hite Model 7008, Brockton, MA, USA), the power amplifier (NF BP4610, Yokohama, Japan), and the multifunction I/O (Input/Output) device (USB-6353, Austin, TX, USA). The physical SST and the measurement system are shown in Figure 1c,d, respectively. The SST consists of two spatially orthogonal yokes, a pending test sample, and a B-H sensor. AC- and DC-biased excitations are sent from the I/O device, amplified by a power amplifier, and applied to the SST. The ***B*** and ***H*** signals detected by the B-H sensor are filtered and amplified by the preamplifier, and then sent back to the I/O device. The AC voltage for rotating magnetic field excitation and the DC voltage for DC-biased field excitation are simultaneously applied to the SST. The valid ***B*** and ***H*** signals, which consist of AC and DC components, respectively, are sensed based on Faraday’s law of induction and the boundary continuity effect of ***H***. The DC components are extracted from the original ***B*** and ***H*** signals via an effective integration and filter process. The pending tested sample is a grain-oriented silicon steel with a square shape of 80 × 80 mm.

### 2.2. The Measured Magnetic Properties of 30ZH120

**Figure 2:**
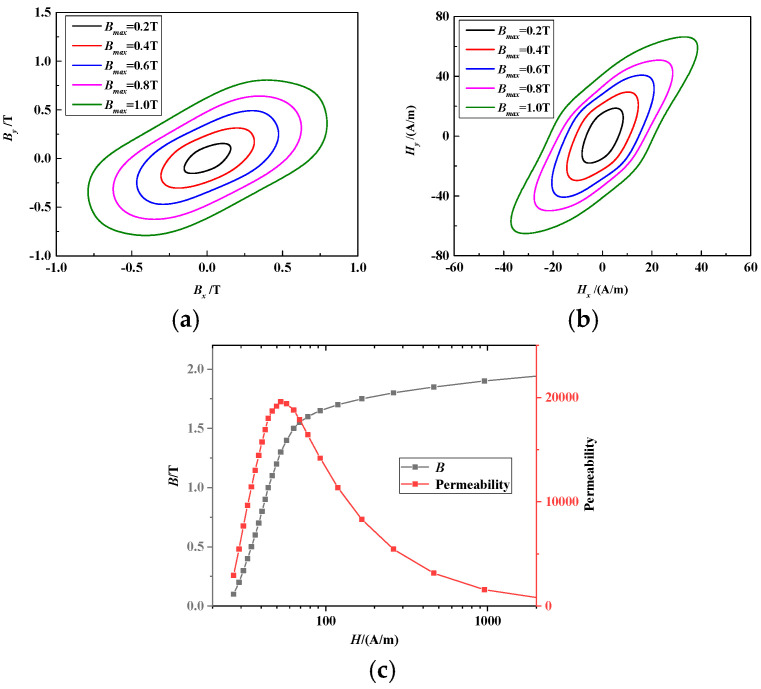
Loci of ***B*** and ***H*** under different ***B_max_*** without DC bias and the corresponding magnetization curve and permeability of 30ZH120: (**a**) ***B*** locus. (**b**) ***H*** locus. (**c**) Magnetization curve and permeability of 30ZH120 at the frequency 50 Hz in easy magnetization direction.

The grain-oriented electrical steel sheet 30ZH120 is commonly utilized in power transformer cores and serves as a test sample in this section. With the influence of different maximum magnetic flux values ***B_max_*** and without DC-biased excitation, the loci of ***B*** and ***H*** are shown in Figure 2a,b, respectively. Here, *B_x_* and *H_x_* are the ***B*** and ***H*** components in the rolling direction, respectively, and *B_y_* and *H_y_* are the ***B*** and ***H*** components in the traverse direction. The ***B*** locus was controlled as an elliptical trajectory, and as ***B_max_*** increased, the amplitudes of *B_x_*, *B_y_*, *H_x_* and *H_y_* also increased. Because of the nonlinear magnetization characteristics of the electrical steel sheet, the ***H*** locus distorts and is not an elliptical shape. The magnetization curve and permeability of 30ZH120 at the frequency 50 Hz in the easy magnetization direction are shown in Figure 2c. As ***H*** increases, ***B*** first rises rapidly and then tends to stabilize. Similarly, the corresponding permeability first increases and then decreases.

**Figure 3:**
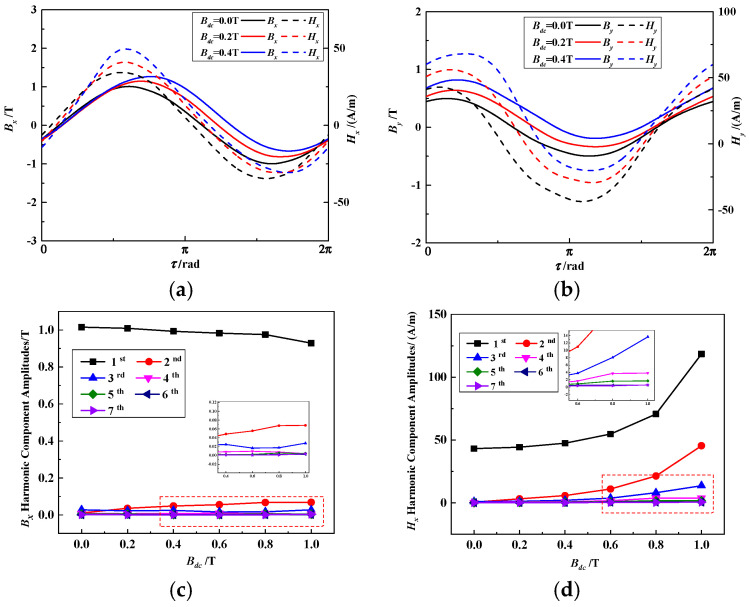
Waveforms and harmonic component amplitudes of ***B*** and ***H*** under different ***B_dc_***: (**a**) *B_x_* and *H_x_* waveforms. (**b**) *B_y_* and *H_y_* waveforms. (**c**) *B_x_* harmonic component amplitudes. (**d**) *H_x_* harmonic component amplitudes.

The waveforms and harmonic component amplitudes of ***B*** and ***H*** under the impact of different DC magnetic flux density amplitudes, ***B_dc_***, are shown in Figure 3. As ***B_dc_*** increases, offset and asymmetric distortion occurs in the *B_x_*, *B_y_, H_x_* and *H_y_* waveforms. Under DC-biased field conditions, the amplitudes of the second harmonic components of *B_x_* increase, whereas the amplitudes of the fundamental, second and third components of *H_x_* increase. The harmonic component of *B_x_* higher than 2nd is less than 0.03 T and is almost noise. The harmonic component of *H_x_* higher than 4th is less than 1 A/m and is almost noise. The harmonic component amplitudes of *B_y_* and *H_y_* have the same trends as those of *B_x_* and *H_x_*. Increasing the harmonic component amplitudes of ***B*** and ***H*** waveforms could lead to a more significant eddy current effect and iron loss.

### 2.3. The Measured Iron Loss

**Figure 4:**
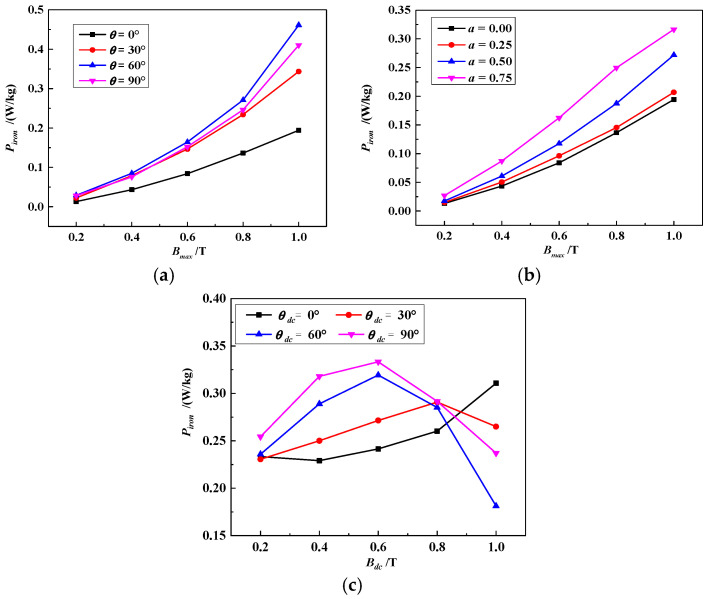
The iron loss under different magnetization conditions: (**a**) The loss of different *B_max_* and *θ*. (**b**) The loss of different *B_max_* and *α*. (**c**) The loss of different *B_dc_*.

Considering vectors ***B*** and vector ***H***, iron core loss can be expressed as follows:

(1) $$P_{iron}=\frac{1}{T_{m}\rho }\int_{0}^{T}H\cdot \frac{dB}{dt}dt=\frac{1}{T_{m}\rho }\int_{0}^{T}(H_{x}\cdot \frac{dB_{x}}{dt}+H_{y}\cdot \frac{dB_{y}}{dt})dt$$

where *P_iron_* represents the iron core loss, *T_m_* represents the magnetization period and *ρ* represents the electrical steel sheet density. The relationship between the loss of grain-oriented silicon steel and magnetization conditions (*B_max_*, *θ*, *α*), as calculated using Equation (1) with a rotating magnetic field and without a DC-biased field, is shown in Figure 4a,b. This indicates that as *B_max_* increases, the loss of the electrical steel sheet *P_iron_* increases. When *θ* = 0°, the overall loss of the electrical steel sheet is relatively small, mainly because 0° is the rolling direction, which is the easy magnetization direction. As α increases, the rotational hysteresis loss of the electrical steel sheets increases, and the corresponding total loss increases. The relationship between the iron loss and the DC bias magnetization conditions (*B_dc_*, *θ_dc_*) is shown in Figure 4c. With the rise of *B_dc_*, except for that of the rolling direction (*θ_dc_* = 0°), the loss in other directions (*θ_dc_* = 30°, 60° and 90°) first increases and then decreases. Moreover, since the directions (*θ_dc_* = 60° and 90°) are difficult to magnetize, the rising and decreasing trend is more pronounced.

**Figure 5:**
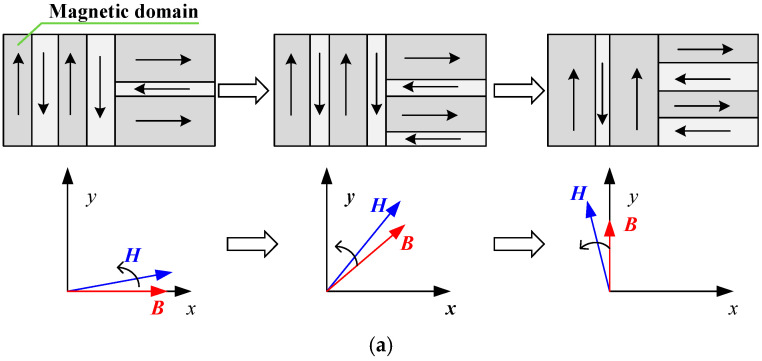

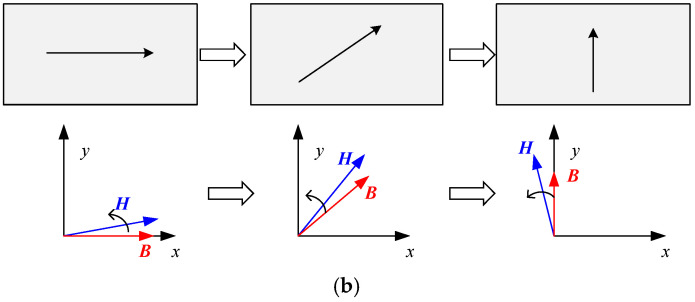
The movement process of magnetic domain: (**a**) The movement process of magnetic domain under the rotating magnetic field increasing for *B_max_* ranging from 0.2 to 1.0 T. (**b**) The movement process of magnetic domain under the rotating magnetic field and DC-biased field for increasing *B_dc_* ranging from 0.2 to 1.0 T and increasing *θ_dc_* from 0 to 90°.

The loss in electrical steel sheets under different magnetization conditions can be further explained from the perspective of the magnetization process, magnetic domain movement and rotation, as shown in Figure 5 [24,25]. The magnetic domain behavior is described in Figure 5a under rotating field conditions for increasing values of *B_max_*, ranging from 0.2 to 1.0 T, and changing values of *θ* and *α*. This description corresponds to the *B*-*P_iron_* shown in Figure 4a,b. Owing to the applied rotating magnetic field direction changing, there are two simultaneous magnetization processes: magnetic domain movement and rotation. The amount of domain movement and rotation depends on the magnitude of the applied magnetic field ***H***. Owing to the pinning of the domain by the defect sites inside the specimen, which causes an opposing force to resist any changes in ***B***, there exists an angle difference between ***H*** and ***B***. With a small rotating magnetic field, there is no disappearance or generation of the domain wall during the movement of the magnetic domain, and the rotational hysteresis loss is small and gradually increases with an increase in *B_max_*. As the applied rotating field increases further, the magnetic domain, which was opposite to the direction of the magnetic field, disappears. As the magnetic field rotates, when the magnetic domain is not opposite the direction of the external magnetic field, it reappears, as shown in Figure 5a. Because the disappearance and generation of magnetic domain walls requires energy, the rotational hysteresis loss is rapid, and the corresponding total loss increases with an increase in *B_max_*. Because the 60° and 90° directions are difficult to magnetize, many domain walls disappear and regenerate when the external magnetic field direction is rotated from 60° to 90°, leading to more iron loss, as shown in Figure 4a. When *α* = 0, the electrical steel sheet is under an alternating magnetic field, and the direction of the external magnetic field does not change, resulting in no rotational hysteresis loss and a relatively small total loss. As *α* increases, the magnetic field direction changes, and more magnetic domain walls in the difficult magnetization direction disappear and are generated again, resulting in an increasing rotational hysteresis loss and corresponding total loss, as shown in Figure 4b. The magnetic domain behavior is described in Figure 5b under rotating and DC-biased fields for increasing *B_dc_* values, ranging from 0.2 to 1.0 T, and increasing *θ_dc_* values from 0 to 90°, which corresponds to the *B*-*P_iron_* relationship of Figure 4c. Under a rotating magnetic field and DC-biased magnetic field, as the amplitude of the external magnetic field further increases (corresponding to an increasing *B_dc_*), more magnetic domain walls disappear and regenerate, leading to an increase in the loss of the electrical steel sheets. When the steel sheet is in a deep saturation state, the wall moves to the boundary interface of the magnetic domain, and the magnetic moment begins to rotate with the applied magnetic field to reduce the lag angle, as shown in Figure 5b. Owing to the fewer magnetic domains at this time, as the amplitude of *B_dc_* increases, the corresponding total loss of the electrical steel sheets decreases. When *θ_dc_* = 0°, the electrical steel sheet is not in the deep saturation state. Hence, the loss increases with the increasing *B_dc_*.

## 3. Enhanced Dynamic Vector Hysteresis Model and Corresponding FEM Calculation

### 3.1. Governing Equation Derivation of the Two-Dimensional Magnetic Field and Temperature Field

The original vector magnetic hysteresis model was proposed to describe the behaviors of ***B*** and ***H*** only under a rotating field [19,20]. The nonlinear relationship between ***B*** and ***H*** can be expressed using an improved dynamic vector hysteresis model, as shown in Equation (2). The total magnetic field strength can be separated into three parts: the reduced magnetic field strength *H_rek_*, the one caused by the eddy current *H_exk_* and the one caused by the DC-biased field *H_dck_*.

(2) $$H_{k}(\tau )=H_{rek}(\tau )+H_{exk}(\tau )+H_{dck}=v_{rk}B_{ack}(\tau )+v_{hk}\int B_{ack}(\tau )d\tau +\frac{\sigma \omega d^{2}}{12}\frac{dB_{k}}{d\tau }+v_{0k}B_{dck}$$

where the time variable *τ* is a dimensionless time normalized by the period which can be expressed from the frequency and range from 0 to 2π; *ω* is the angular frequency of 50 Hz; *σ* is the resistivity; *B_ack_* is the AC component of the ***B*** waveform; *B_dck_* is the DC component of the ***B*** waveform; *d* is the thickness of the sheet, where the subscript *k* indicates *x* or *y*, which means that the parameters with the subscript *k* represent the corresponding components on the *x*- or *y*-axis. *v_rk_* is the rotating reluctivity coefficient, *v_hk_* is the rotating hysteresis coefficient and *v*_0*k*_ is the DC reluctivity coefficient. The *H_rek_* and *H_exk_* terms can be expressed using the following equations [21,22]:

(3) $$H_{rek}(\tau )=v_{rk}B_{ack}(\tau )+v_{hk}\int B_{ack}(\tau )d\tau$$

(4) $$H_{exk}(\tau )=\frac{\sigma \omega d^{2}}{12}\frac{dB_{k}}{d\tau }$$

By combining the improved model with Maxwell’s equations, the governing equation of the two-dimensional magnetic field can be derived as follows:

(5) $$\nabla \times v_{r}(\nabla \times A_{ac})+\nabla \times v_{h}\int (\nabla \times A_{ac})d\tau +\nabla \times \frac{\sigma \omega d^{2}}{12}\frac{\partial (\nabla \times A_{ac})}{\partial \tau }+\nabla \times v_{0}(\nabla \times A_{dc})=J$$

where ***A_ac_*** is the AC component of the magnetic vector potential, ***A_dc_*** is the DC component of the magnetic vector potential and ***J*** is the excitation current density. Finite element discretization can be performed on the solution domain Ω*_e_*, and Equation (5) can thus be re-written as follows:

(6) $$\begin{array}{l} \sum_{E}\left[\iint_{\Omega_{e}}\left(\frac{\partial A_{ac}^{e}}{\partial y}\cdot v_{rx}\frac{\partial W^{e}}{\partial y}+\frac{\partial A_{ac}^{e}}{\partial x}\cdot v_{ry}\frac{\partial W^{e}}{\partial x}\right)dxdy\right]\\ \;\;+\sum_{E}\left\{\int \left[\iint_{\Omega }\left(\frac{\partial A_{ac}^{e}}{\partial y}\cdot v_{hx}\frac{\partial W^{e}}{\partial y}+\frac{\partial A_{ac}^{e}}{\partial x}\cdot v_{hy}\frac{\partial W^{e}}{\partial x}\right)dxdy\right]d\tau \right\}\\ \;\;\;+\sum_{E}\left\{\frac{\sigma \omega d^{2}}{12}\frac{\partial }{\partial \tau }\left[\iint_{\Omega }\left(\frac{\partial A_{ac}^{e}}{\partial y}\frac{\partial W^{e}}{\partial y}+\frac{\partial A_{ac}^{e}}{\partial x}\frac{\partial W^{e}}{\partial x}\right)dxdy\right]\right\}\\ \;\;\;\;+\sum_{E}\left[\iint_{\Omega_{e}}\left(\frac{\partial A_{dc}^{e}}{\partial y}\cdot v_{0x}\frac{\partial W^{e}}{\partial y}+\frac{\partial A_{dc}^{e}}{\partial x}\cdot v_{0y}\frac{\partial W^{e}}{\partial x}\right)dxdy\right]=\sum_{E}\left(\iint_{\Omega }W^{e}J^{e}dxdy\right) \end{array}$$

where *W^e^* is the discretized weight function, the superscript *e* represents a single element and *E* is the total number of elements. The magnetic field distribution can be calculated by solving Equation (6). For a three-leg three-phase transformer core, by integrating the dynamic vector hysteresis model with finite element analysis, the distribution of magnetic parameters within the transformer core under a DC-biased field can be calculated. In this study, a DC-biased current was applied to the transformer winding, which implied that a DC-biased magnetic field was applied to the transformer core. The parameters (*B_max_*, *α*, *θ*, *B_dc_* and *θ_dc_*) of rotating and DC-biased fields in each element are updated during each iteration of the FEM calculation. The corresponding *v_r_*, *v_h_* and *v*_0_ in Equation (6) also change according to different parameters (*B_max_*, *α*, *θ*, *B_dc_* and *θ_dc_*) during each iteration. Furthermore, a magnetization curve was introduced into the finite element analysis for comparison. The magnetic field distribution and the corresponding iron loss distribution are calculated in both cases: in which the magnetization curve is used and that in which an improved model is used.

The temperature rise caused by the winding loss can be conducted to the transformer core and exacerbates the local overheating problem under a DC bias. Therefore, in addition to the iron core, the copper loss from the winding generated under the DC-biased excitation should also be considered. The relationship between the copper loss and the current density *J* can be expressed as follows:

(7) $$P_{coil}=\frac{J^{2}}{\rho_{coil}\sigma_{coil}}$$

where *P_coil_* and *ρ_coil_* are the copper loss and density of the winding, respectively, and *σ_coil_* represents the conductivity of copper. *P_coil_* is included in both cases: in the case when the magnetization curve is used and in the case when an improved model is used.

Owing to the solid domain of the iron core and winding, the heat dissipation method involves heat conduction. The thermal convection effect of air in the calculation domain is ignored. According to Fourier’s law and the energy conservation law, under DC bias magnetization, the local loss and temperature change in the transformer iron core model satisfy the heat conduction governing equation, as follows:

(8) $$\frac{\partial T}{\partial t}=\frac{\lambda }{\rho C}\left(\frac{\partial^{2}T}{\partial x^{2}}+\frac{\partial^{2}T}{\partial y^{2}}\right)+\frac{P}{C}$$

where *P* represents the loss of iron core or winding, *C* indicates the specific heat capacity, *λ* is the heat transfer coefficient and *T* represents the temperature.

The space discretization is performed in the solution domain Ω*_e_*, where Equation (8) could be expressed as follows:

(9) $$\begin{array}{l} \sum_{E}\left\{\iint_{\Omega_{e}}\left[\frac{\partial W^{e}}{\partial x}\left(\frac{\lambda }{\rho }\frac{\partial T^{e}}{\partial x}\right)+\frac{\partial W^{e}}{\partial y}\left(\frac{\lambda }{\rho }\frac{\partial T^{e}}{\partial y}\right)\right]dxdy\right\}\\ \;\;\;\;\;\;\;\;\;\;\;\;+\sum_{E}\left[\iint_{\Omega_{e}}W^{e}\left(C\frac{\partial T^{e}}{\partial t}\right)dxdy\right]=\sum_{E}\left(\iint_{\Omega_{e}}W^{e}P^{e}dxdy\right) \end{array}$$

where *T_e_* is the temperature of each element and *P_e_* is the loss of each element.

### 3.2. Analysis of the Magnetic Properties Distribution

**Figure 6:**
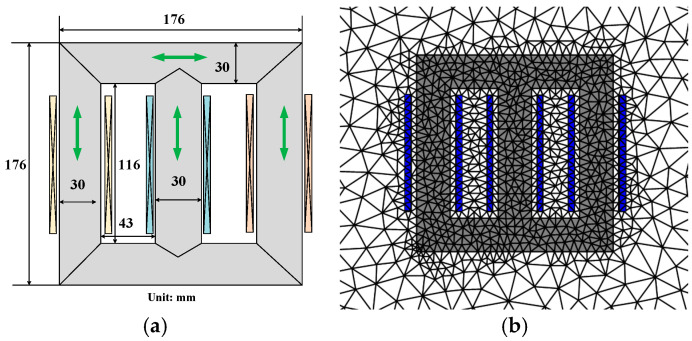
Transform core model and the mesh: (**a**) The transform core model. (**b**) The mesh.

A three-phase transformer core model was established for the FEM calculations under rotating and DC-biased fields. The transformer core model was made of multiple laminated, oriented 30ZH120 electrical steel sheets, ensuring that the direction of the iron yokes was parallel to the rolling directions of the electrical steel sheet, as shown in Figure 6a. The arrows indicate the rolling direction of electrical steel sheets. An AC excitation voltage with an amplitude of 3 V and a phase angle difference of 120° is applied to the three-phase winding, and a DC excitation voltage with an amplitude of 0.2 V is simultaneously applied to the left and right windings. The 2D automatic mesh with triangular elements on the three-phase transformer core model is shown in Figure 6b. The nonlinear governing Equations (6) and (9) were solved using the LinearSolve method. The FEM calculations were performed under Dirichlet boundary conditions.

**Figure 7:**
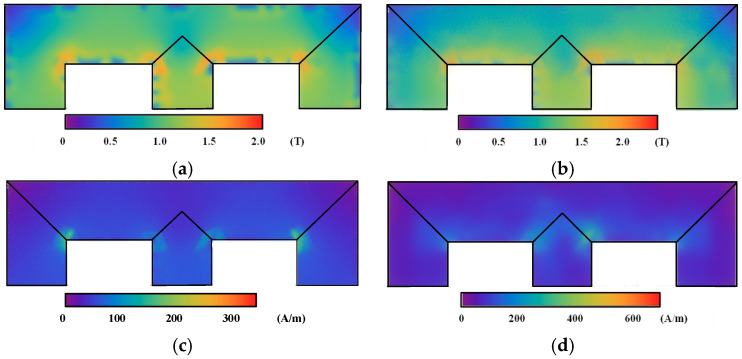
Distribution of the maximum value of ***B*** and ***H*** of the transformer core model: (**a**) Maximum value of ***B*** calculated using the magnetization curve. (**b**) Maximum value of ***B*** calculated using the improved model. (**c**) Maximum value of ***H*** calculated using the magnetization curve. (**d**) Maximum value of ***H*** calculated using the improved model.

Figure 7 illustrates the maximum values of ***B*** and ***H*** under DC-biased and rotating fields, respectively. The maximum value of ***B*** occurred at the upper window edge of the iron core, which was caused by the concentration of the magnetic force line. Compared with those of magnetization curve, the maximum value of ***H*** in the improved model was 251 A/m and was larger than that of the magnetization curve (173 A/m). This is because the hysteresis and eddy current effects of the rotating magnetic field are neglected in the FEM calculation of the magnetization curve.

### 3.3. Analysis of Core Loss and the Temperature

**Figure 8:**
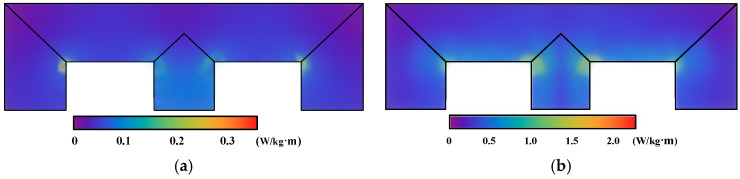
Distribution of iron loss of the transformer core model: (**a**) Iron loss calculated using the magnetization curve. (**b**) Iron loss calculated using the improved model.

Figure 8 shows the distribution of the iron core loss under DC bias magnetization, as calculated using the magnetization curve and dynamic vector hysteresis model. The maximum loss calculated by the magnetization curve is 0.321 W/kg·m, and that calculated by the dynamic model is 1.652 W/kg·m. Owing to the influence of the rotating magnetic field and the hysteresis effect of the electrical steel sheet, compared with the magnetization curve, the core loss under DC bias, as calculated by the dynamic vector hysteresis model, has larger values at the corners and T-joint area of the transformer core model.

**Figure 9:**
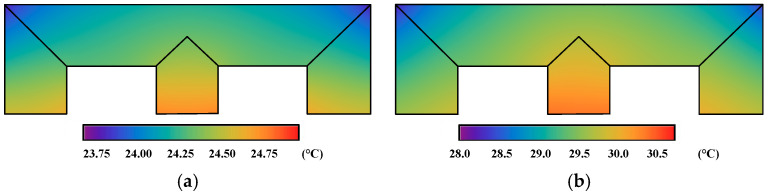
Distribution of temperature of the transformer core model: (**a**) Temperature calculated using the magnetization curve. (**b**) Temperature calculated using the improved model.

The uneven distribution of the iron core losses in Figure 8 and the winding loss were applied as the heat source load, and the temperature distribution of the transformer iron core model under DC bias was calculated using the magnetization curve and dynamic vector hysteresis model, as shown in Figure 9. The original temperature is the room temperature, 20 °C. Owing to the impacts of the copper loss of the winding and the heat conduction effect, the hottest point of the iron core model was near the middle core limb position.

The corner temperature calculated by the magnetization curve increases by 4.29 °C, and the T-joint area increases by 4.41 °C. At the same position, the corner temperature calculated by the dynamic model increases by 9.60 °C, and the T-joint area increases by 9.91 °C. The overall temperature calculated by the dynamic model is about 5 °C higher than that calculated by the magnetization curve. This is mainly because the dynamic model considers the influence of the rotating magnetic field and hysteresis effect, resulting in a higher calculated iron core loss. Additionally, compared with those in the rolling direction, the values of ***H***, iron loss and temperature calculated by the magnetization curve in traverse direction would be larger, since the steel sheet is harder to magnetize in this direction. The magnetization curve in a different direction would be applied in the FEM calculation and would be further analyzed in follow-up research.

## 4. Verification of the FEM Calculation

To verify the reliability of the FEM calculation, the temperature distribution test results for a 110 kV single-phase autotransformer power transformer under a DC bias are utilized as comparative data. The measured excitation current is applied as the input for the numerical analysis, and the distribution of the magnetic parameters in the power transformer core under a DC bias is calculated. Furthermore, the iron loss and temperature increase are calculated and compared with the measurement results of the DC bias test of the physical power transformer. The single-phase transformer is highly sensitive to DC magnetic flux. Therefore, 110 kV single-phase autotransformer power transformer is selected as the tested subject. The main technical parameters of the tested transformer are listed in Table 1.

**Figure 10:**
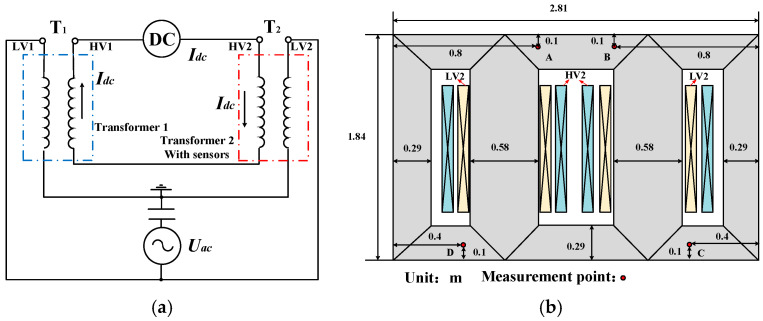
Schematic diagram of DC bias test: (**a**) Wiring schematic diagram for DC bias test. (**b**) Temperature rise measurement point for DC bias magnetic test.

The connection mode of the DC magnetic bias test for power transformers is illustrated in Figure 10a. Two 110 kV power transformers are connected in parallel on the high-voltage side, and the low-voltage side is also connected in parallel. The transformer is equipped with sensors to measure the local temperature increase. Transformer 1 does not contain sensors and is tapped negatively, whereas Transformer 2 is tapped positively. Owing to the different turns of the high-voltage winding, there is a circulating current on the low-voltage side, which means that Transformer 2 is in operation state. The DC bias excitation is introduced from the high-voltage side winding of the transformer, so that Transformer 2 operates in a DC-biased state.

**Figure 11:**
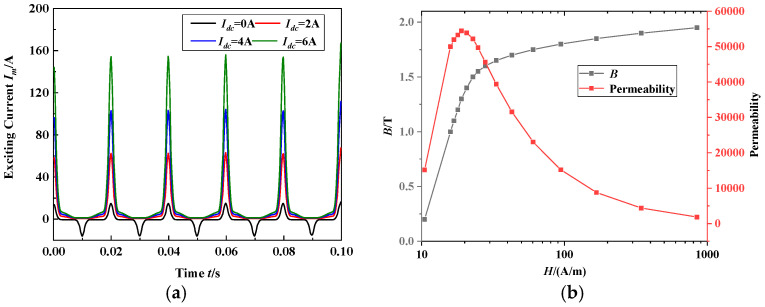
Exciting current and magnetization curve of the tested transformer core: (**a**) Exciting current for different DC bias conditions. (**b**) Magnetization curve and the corresponding permeability of 27RK090 (50 Hz) of the tested transformer core in easy magnetization direction.

The transformer structure is shown in Figure 10b. The windings on the high-voltage (HV2) and low-voltage (LV2) sides are mounted around the two main limbs. T-type thermocouples are embedded in the T-joint area on the surfaces of the two main limbs of the transformer core to measure the local temperature increase under DC bias magnetization, since this area is more sensitive to the rotating magnetic field. The original temperature is the room temperature, 20 °C. The currents of the high- and low-voltage windings of the transformer are measured, and the corresponding excitation current is obtained, as shown in Figure 11a. The exciting current in Figure 11a is directly introduced into the transformer core in the FEM calculation to simulate the DC-biased situation of different *I*_dc_ values (*I*_dc_ = 0, 2, 4 and 6 A). The core of the 110 kV single-phase autotransformer power transformer is made of a grain-oriented 27RK090 electrical steel sheet. The magnetization curve and corresponding permeability of 27RK090 of the frequency 50 Hz in the easy magnetization direction are shown in Figure 11b. The experimental data are presented in [26].

**Figure 12:**
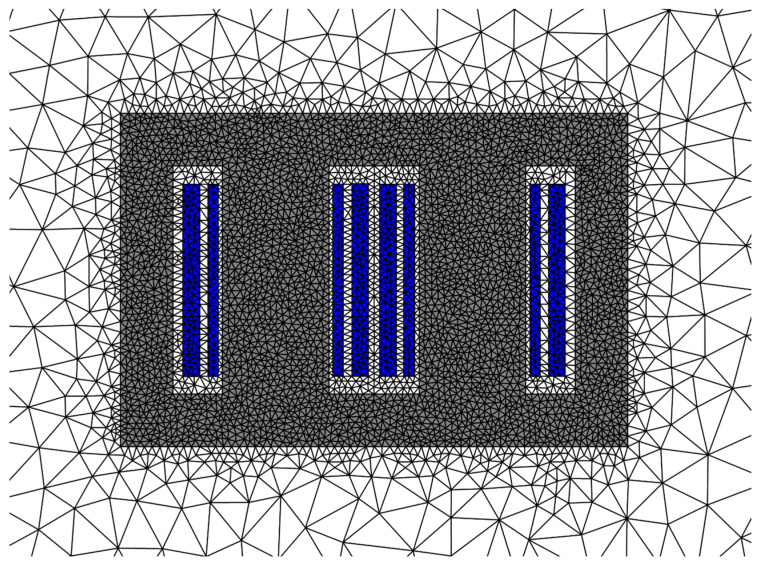
The 2D automatic mesh with triangular elements of the physical transformer core.

The 2D automatic mesh with triangular elements is applied to the physical transformer core, as shown in Figure 12. The magnetic property distribution and corresponding loss and temperature rise distributions can be obtained by solving the nonlinear governing Equations (6) and (9) via the LinearSolve method.

**Figure 13:**
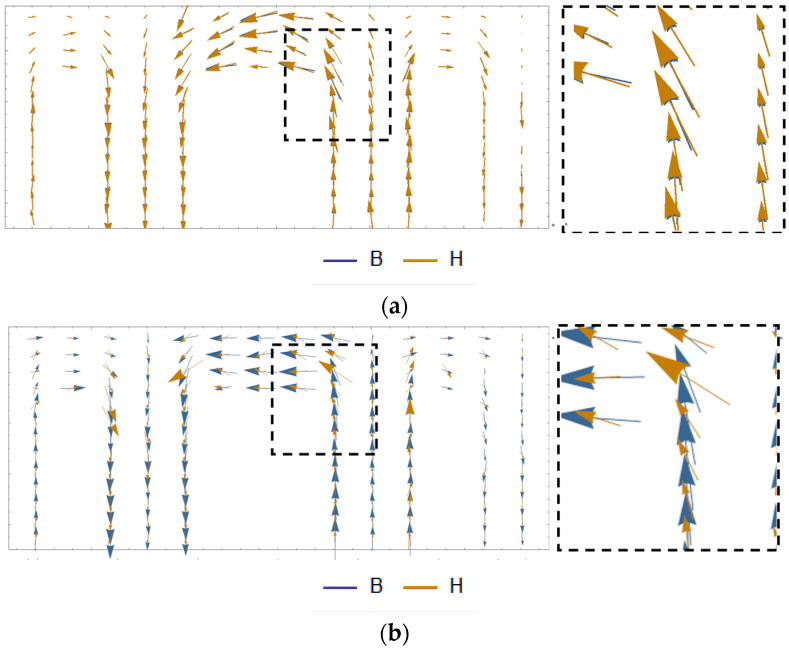
Distribution of ***B*** and ***H*** of the physical 110 kV transformer core: (**a**) Distribution of ***B*** and ***H*** calculated using the basic magnetization curve. (**b**) Distribution of ***B*** and ***H*** calculated using proposed model.

The distributions of ***B*** and ***H*** calculated using the dynamic model and the basic magnetization curve are shown in Figure 13, where *I_dc_* = 6 A. For the magnetization curve, ***B*** is parallel to ***H***, but there is an angle lag between the calculated ***B*** and ***H*** of the dynamic model at the T-shaped connection and corner of the transformer core, which is the characteristic of the rotating magnetic field that is considered in the proposed model.

**Figure 14:**
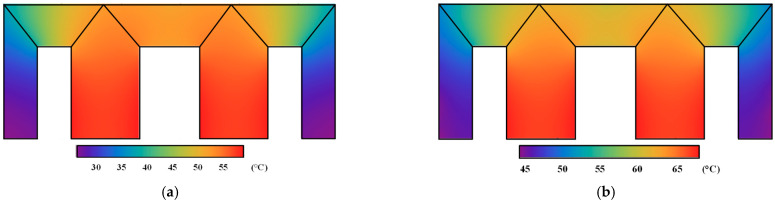
Distribution of temperature of the physical 110 kV transformer core: (**a**) Temperature calculated using the magnetization curve. (**b**) Temperature calculated using the improved model.

The temperature distribution of the transformer iron core with *I_dc_* = 6 A is calculated using the magnetization curve and dynamic vector hysteresis model, as shown in Figure 14. These results indicate that the maximum temperature occurred in the main limb area of the transformer core, which is related to the heat conduction of the winding. Compared with the magnetization curve, the temperature of the proposed model considering the hysteresis and eddy current effects has a larger value.

To verify the local temperature rise of the transformer core at points A, B and C under different *I*_dc_ values (*I*_dc_ = 0, 2, 4 and 6 A), the waveforms of the vector magnetic flux density and vector magnetic field intensity in a magnetization period and the corresponding core loss are calculated using the dynamic vector hysteresis model and basic magnetization curve under a DC bias, as shown in Table 2.

As *I*_dc_ increases, the half-wave saturation in the transformer core becomes more pronounced, which causes the ***H*** waveform to peak, indicating a stronger hysteresis effect. Consequently, the corresponding local losses at points A, B, C and D, as calculated using the dynamic model and magnetization curve, also increase. Points A and B are situated in the main magnetic circuit of the transformer core, whereas points C and D are in the iron core bypass. The magnitudes of ***B*** and ***H*** in the main magnetic circuit are higher than those in the bypass circuit. Therefore, the calculated loss values of points A and B of the dynamic model and the magnetization curve are higher than the those of points C and D. Considering the hysteresis and eddy current effects of rotating magnetic field and DC-biased field in the dynamic model, the local loss value of the proposed model is larger than that of the magnetization curve.

**Figure 15:**
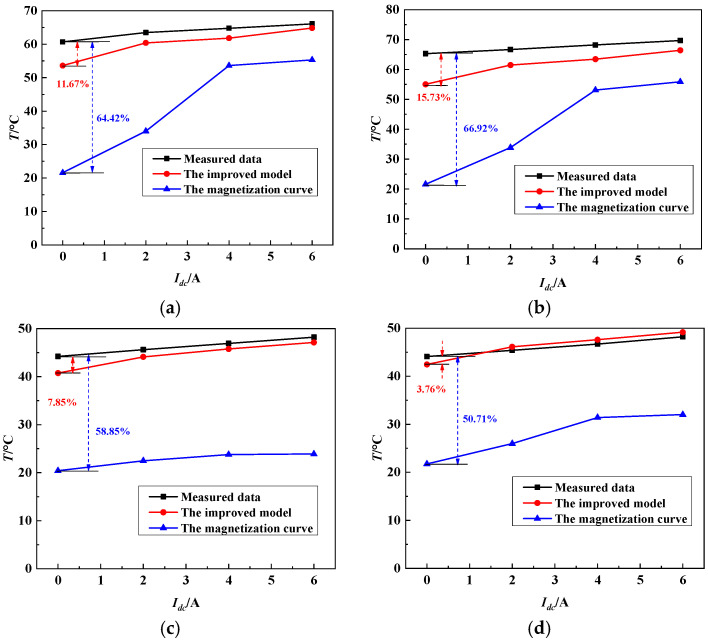
Comparison between calculated and measured values of local temperature of iron core: (**a**) Point A. (**b**) Point B. (**c**) Point C. (**d**) Point D.

To verify the finite element calculation results of the power transformer core considering the dynamic vector hysteresis model under a DC magnetic bias, it is necessary to compare the temperature changes at points A, B, C and D under different DC bias conditions with the measured data, as shown in Figure 15. During the DC bias test, different *I_dc_* values are applied to the transformer for 2 h to measure stable and accurate temperature values using a T-type thermocouple. The calculated results are obtained by combining the finite element analysis with the magnetization curve and using the dynamic model and the magnetization curve. As *I*_dc_ increases, the transformer core experiences the half-wave saturation, thereby leading to an increase in core losses. Consequently, the calculated temperatures at points A, B, C and D of both the magnetization curve and the dynamic model increase. The temperatures at points A and B are higher than those at points C and D. This is because ***B*** and ***H*** in the main magnetic circuit are higher than those in the bypass circuit. Because the hysteresis effect and anisotropy of the transformer core under a rotating magnetic field and DC-biased field are considered in the improved model, the local temperature calculated by the dynamic model is higher than that calculated by the magnetization curve, and is more consistent with the measured data. The calculated errors of the temperature increase at points A, B, C and D are listed in Table 3. The calculation error of the proposed model ranges from 3.76 to 15.73%, which is far less than the error of the magnetization curve (approximately 50.71–66.92%). These results demonstrate that the FEM calculation results of the dynamic vector hysteresis model, which considers the impact of hysteresis and eddy current effects under a DC bias, can provide more accurate temperature estimations than those of the basic magnetization curve.

To simplify the FEM analysis, only the influences of the iron core loss and winding copper loss are considered in this study, and the influence of the stray loss of the metal structural components is neglected in the temperature rise calculation. Therefore, the calculation error of the proposed model reaches a maximum value of 15.73%. The impact of the stray loss of the metal structural components on the temperature rise of the transformer core will be discussed in further research. In addition, because of the difficulty in measuring the local vector magnetic parameters and losses of the transformer cores, only the local temperature rise is calculated and verified using the experimental data in this study. An improved method for the local magnetic parameters detection of the transformer core will be studied, and the FEM results calculated by the proposed vector model will be further verified in future research.

Owing to the large volume and high voltage levels of actual ultrahigh-voltage transformers, the amplitude of the current flowing through the neutral point of the winding under a DC bias is large. Therefore, compared with the transformer tested in this study, the local overheating temperature of ultrahigh-voltage transformers under a DC bias is higher, and further DC bias isolation protection in the power network is indispensable.

## 5. Conclusions

In this study, an improved dynamic vector hysteresis model that considers the hysteresis and eddy current effects was introduced into the FEM analysis of the iron loss and temperature distribution of a transformer core under a DC-biased field. A measurement system for the magnetic characteristics under rotating and DC-biased fields was established, and the iron loss was measured under different DC-biased magnetization conditions. As the magnetic domain vanished, regenerated and moved, the iron loss first increased and then decreased with the increasing *B_dc_*. An enhanced model was established by introducing reduced magnetic field strengths, one caused by eddy current and one caused by a DC-biased field. The proposed model was combined with a numerical analysis to simulate the distribution of magnetic properties, iron loss and temperature. The maximum values of the ***H*** and the iron loss occurred at the corners and T-joint of the core, and the temperature was higher in the main limb area. The FEM calculation results were verified using the temperature rise data of the 110 kV power transformer. Compared with the magnetization curve, the temperature distribution of the proposed model, with a calculation error ranging from 3.76 to 15.73%, was more accurate and reliable. The calculation error would be minimized with consideration of the stray loss of metal structural components in further research. The local temperature of a transformer core can exceed 65 °C when *I_dc_* = 6 A, indicating that DC bias isolation protection is indispensable in the power network.

## Figures and Tables

**Table 1:** The main technical parameters of the tested transformer.

Technical Parameters		Value	Technical Parameters		Value
Electrical parameters	Rated voltage	$110/\sqrt{3}\pm 10\%/10.5\mathrm{kV}$	Low-voltage side winding	Winding method	Continuous
	Rated capacity	21 MVA		Number of turns	112
High-voltage side winding	Winding method	Sandwich-interleaved	Iron core	Brand	27RK090
	Number of turns	583~729		Structure	Single-phase four-limb

**Table 2:** Local losses of transformer core at Points A, B, C and D.

Core Loss P/(W/kg)	Point A		Point B		Point C		Point D	
	Dynamic Model	Magnetization Curve	Dynamic Model	Magnetization Curve	Dynamic Model	Magnetization Curve	Dynamic Model	Magnetization Curve
*I_dc_* = 0 A	2.092	0.099	2.179	0.100	1.290	0.025	1.182	0.020
*I_dc_* = 2 A	2.514	0.871	2.582	0.862	1.501	0.154	1.523	0.141
*I_dc_* = 4 A	2.602	2.092	2.704	2.062	1.602	0.234	1.716	0.251
*I_dc_* = 6 A	2.791	2.198	2.888	2.233	1.687	0.242	1.762	0.269

**Table 3:** The calculated error of temperature rise at Points A, B, C and D.

Calculated Error	Point A		Point B		Point C		Point D	
	Dynamic Model	Magnetization Curve	Dynamic Model	Magnetization Curve	Dynamic Model	Magnetization Curve	Dynamic Model	Magnetization Curve
*I_dc_* = 0 A	11.67%	64.42%	15.73%	66.92%	7.85%	53.84%	3.76%	50.71%
*I_dc_* = 2 A	4.88%	46.45%	7.81%	49.23%	3.28%	50.72%	1.56%	42.81%
*I_dc_* = 4 A	4.60%	17.25%	6.95%	22.08%	2.45%	49.33%	1.92%	32.78%
*I_dc_* = 6 A	1.89%	16.29%	4.717%	19.81%	2.26%	50.45%	1.98%	33.58%

## Data Availability

The raw data supporting the conclusions of this article will be made available by the authors on request.
